# Role of Anions in the Synthesis and Crystal Growth of Selected Semiconductors

**DOI:** 10.3389/fchem.2022.881518

**Published:** 2022-04-25

**Authors:** Shaidatul Najihah Matussin, Ashmalina Rahman, Mohammad Mansoob Khan

**Affiliations:** Chemical Sciences, Faculty of Science, Universiti Brunei Darussalam, Gadong, Brunei

**Keywords:** semiconductors, metal oxides, chalcogenides, shaped-dependent properties, Anion directed crystal growth

## Abstract

The ideal methods for the preparation of semiconductors should be reproducible and possess the ability to control the morphology of the particles with monodispersity yields. Apart from that, it is also crucial to synthesize a large quantity of desired materials with good control of size, shape, morphology, crystallinity, composition, and surface chemistry at a reasonably low production cost. Metal oxides and chalcogenides with various morphologies and crystal structures have been obtained using different anion metal precursors (and/or different sulfur sources for chalcogenides in particular) through typical synthesis methods. Generally, spherical particles are obtained as it is thermodynamically favorable. However, by changing the anion precursor salts, the morphology of a semiconductor is influenced. Therefore, precursors having different anions show some effects on the final forms of a semiconductor. This review compiled and discussed the effects of anions (NO_3_^−^, Cl^−^, SO_4_^2-^, CH_3_COO^−^, CH(CH_3_)O^−^, etc.) and different sources of S^2-^ on the morphology and crystal structure of selected metal oxides and chalcogenides respectively.

## Introduction

Heterogeneous photocatalysis using semiconductors has drawn attention following the discovery of the Honda-Fujishima effect in 1972 (Liu et al., 2014). Photocatalysis has also gained remarkable attention due to its potential application for energy shortage and environmental issues which include hydrogen production from water (Shi et al., 2019), carbon dioxide reduction to fuels (M.S et al., 2021), and toxic pollutants removals in the environment (Koutavarapu et al., 2021). Semiconductor consists of a band structure in which the conduction band (CB) is separated from the valence band (VB) by a band gap. This is one of the important properties as it determines the light absorption and the redox capabilities of a semiconductor. Theoretically, in photocatalysis, when the energy of incident light is equal or larger than that of the band gap of a semiconductor, electrons (e^−^) and holes (h^+^) are generated in the CB and VB, respectively (Matussin et al., 2020a; Naidi et al., 2021; Rahman et al., 2021). These photogenerated charge carriers may be involved in the following possible processes:

1) Migrate to the surface-active sites of semiconductor
2) Captured by the defect sites in bulk and/or on the surface of the semiconductor
3) Recombine and release energy in the form of heat or photon

The last two processes are, however, considered to be deactivation processes due to these photogenerated e^−^ and h^+^ would not involve in photocatalytic reactions. A large number of inorganic semiconductors have been explored including metal oxides, IV group, III-V compounds, and metal chalcogenides. Semiconductor oxide nanomaterials-based photocatalysts have been recognized as one of the most promising areas of research and application such as TiO_2_, ZnO, SnO_2_, CeO_2_, etc (Qi et al., 2013; Khan et al., 2017; Kowsari et al., 2017; Parwaiz et al., 2019; Matussin et al., 2020b; Rahman and Khan, 2021). Metal oxides are mainly used as photocatalysts due to their non-toxicity, low cost, stability, and resistance to photocorrosion.

In comparison to most of the metal oxides, semiconductors including III-V compounds, IV group, and metal chalcogenides show narrow band gap, large optical absorption coefficients, and broad-spectrum light collection (Popescu, 2006; Ahluwalia, 2017). They are called narrow-gap semiconductors in which the band gap of these semiconductors is usually less than 2.3 eV. This could allow light absorption at the wavelength of more than 540 nm. Chalcogenides are compounds consisting of at least one chalcogen anion (S^2-^, Se^2-^ or Te^2-^) and at least one electropositive element. Chalcogenides have drawn significant attention due to their great and highly demanded properties including narrow band gap energy, non-toxicity, and bio-compatibility.

The performance of a semiconductor is strongly correlated with its size. When the size of materials falls into the nanoscale, materials may exhibit different properties (Navya and Daima, 2016). As the size is reduced, the atoms or ions percentage exposed on the surface increases, resulting in an increase in the surface to volume ratio (Navya and Daima, 2016). Therefore, the number of active sites for catalytic reactions increases. Moreover, the reduction of size might also affect the electronic properties of the material. In particular, as the material size is smaller than its Bohr radius, the movement of the charge carriers is greatly confined in physical size due to the quantum confinements. This results in the discrete electronic band structure, leading to size-dependent electronic and optical properties (Li and Wu, 2015).

Furthermore, the morphology of a catalyst is crucial since factors such as the size and shape of particles, the energy associated with facets, coordination of atoms, and the presence of protective ligands can mainly influence its catalytic efficiency (Cao et al., 2016; Guo et al., 2018; Lin et al., 2019; Mishra and Nanda, 2020). In a recent study, Chiu et al. (2012) conducted facet-dependent catalytic activity of Au nanocubes, octahedral, and rhombic dodecahedra towards 4-nitroaniline. It was reported that anisotropic shape particles can alter the reaction performance due to differences in crystal facets exposed. Therefore, the concept of morphology-dependent catalytic and/or photocatalytic activity of a semiconductor has become a growing topic in catalysis and for the exploration of potential applications nowadays.

Varied shapes and sizes of semiconductors are reported to have been obtained through different synthesis methods for instance hydrothermal, precipitation, sol-gel, microwave, green synthesis, and many others (Qi et al., 2013; Sahay et al., 2013; Soren et al., 2015; Hasnidawani et al., 2016; Yin et al., 2016). Furthermore, counter-anion in the metal salts precursors plays a role in the shape-selective growth of semiconductor nanomaterials. It is said that the inorganic anions themselves might be selectively adsorbed on particular facets and thus greatly affect the size, and morphology of the nanomaterials (Herricks et al., 2004; Qi et al., 2014). To date, the lack of studies on anions effects on the development of metal oxides and chalcogenides have become a challenge to prepare metal oxides and chalcogenides with controlled morphology and size. Moreover, various shaped semiconductors without implementation of agents are somehow in demand to prevent high-cost methods and chemical hazards. Recently, researchers have gradually begun studies on the effects of anions on the production of semiconductors. Therefore, in this review, different morphologies of metal oxides and chalcogenides obtained using different metal salts precursors and their crystal growth are discussed in-depth. To the authors’ knowledge, there has been no review on the development of semiconductors using different metal precursors having different anions. This is the first review and compilation of the role of anions in the synthesis and crystal growth of selected metal oxides and chalcogenides.

## Anion Directed Synthesis of Metal Oxides

Metal oxides nanoparticles (NPs) have been widely exploited for many different areas such as toxic pollutants removal (Gowthaman et al., 2020; Yang et al., 2020; Zhou et al., 2021), drug delivery (He et al., 2019; Mallakpour et al., 2022), hydrogen production (Chen et al., 2015; Bhosale et al., 2016; Chen et al., 2018), CO_2_ reduction (Loh and Kherani, 2019; Sun et al., 2021; Kuan et al., 2022), optoelectronics (C. Nehru et al., 2012; Jayakumar et al., 2022; Wang et al., 2010), etc., Controllable growth of metal oxides NPs with defined morphology such as spherical, rod-like, sheet-like, cubic amongst others have been synthesized and reported to have an influence on their catalytic properties.

**FIGURE 1:**
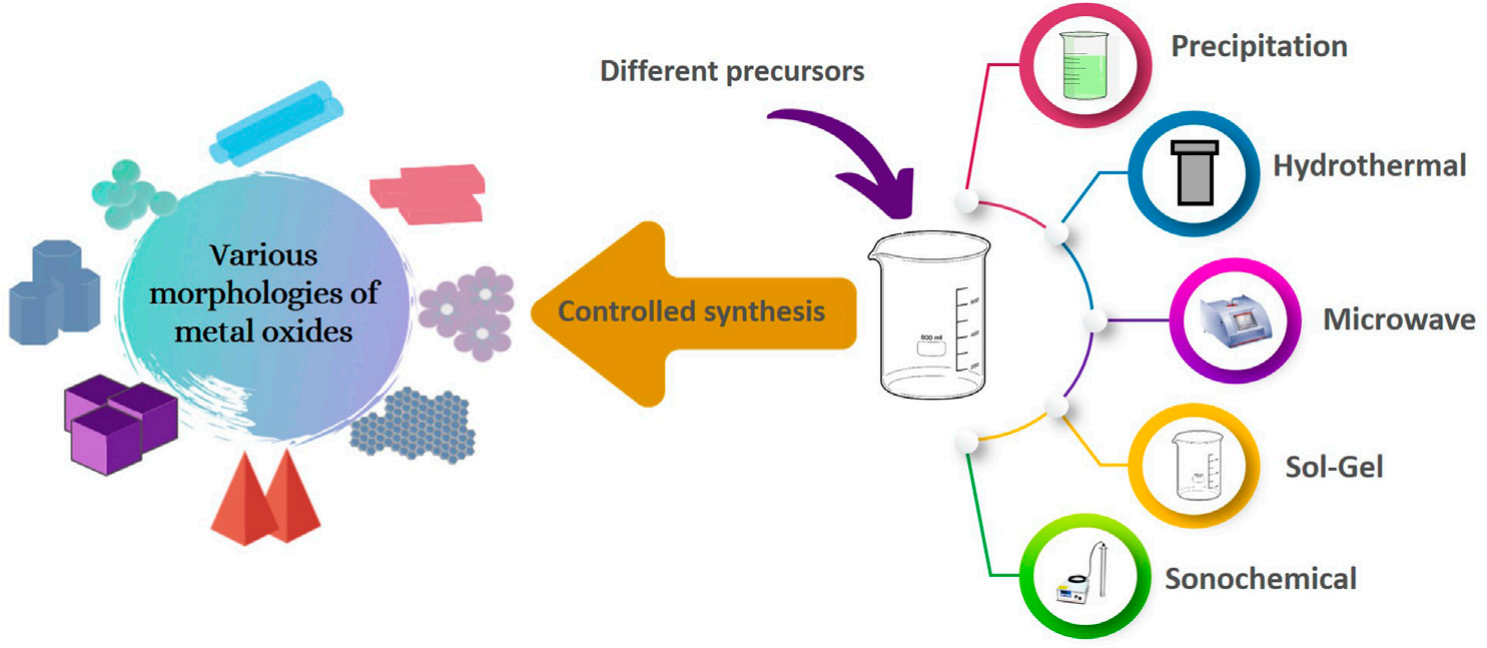
Different morphologies of metal oxides synthesized using different precursors having different anions.

Various morphologies of metal oxides have been acquired from different metal precursors salts (Figure 1). For instance, Panda *et al.* synthesized ZnO nanorods through a sonochemical method using two different Zn precursors namely: Zn(CH_3_COO)_2_ and Zn(NO_3_)_2_·6H_2_O dissolved in a basic condition at room temperature using ammonium acetate and ammonia solution (Panda et al., 2013). Flower-like ZnO was obtained when NO_3_^−^ anion precursor was used, while CH_3_COO^−^ anion precursor showed a nanorod with an average width size between 150 and 500 nm for both anions. Similarly, Gusatti et al. (2011) prepared ZnO *via* the sonochemical method. However, Zn(NO_3_)_2_·6H_2_O and ZnCl_2_ were used. NaOH was added to both the solutions at 90°C resulting in a mixture of short nanoprisms and nanorods of 18.91 nm long and 11.50 nm wide for NO_3_^−^ anion precursor and nanorods of 23 nm diameter for Cl^−^ anion precursor. High purity ZnO NPs were synthesized using Zn(NO_3_)_2_·6H_2_O, Zn(CH_3_COO)_2_, ZnSO_4_·7H_2_O and ZnCl_2_ using a typical precipitation method as reported by Pourrahimi et al. (2014) The precursors’ solutions were stirred at 60°C for 15 min and pre-heated NaOH was added to the solutions yielding star-shaped particle (500 nm) for NO_3_^−^ anion, cone-shaped particle (25 nm) for CH_3_COO^−^ anion, petal-like for both SO_4_^−^ and Cl^−^ anions (80–100 nm).

The formation of hexamethylenetetramine (HMTA)-mediated ZnO nanoparticles was reported by van Rijt et al. (2020) The ZnO particles were synthesized using Zn(NO_3_)_2_·6H_2_O, Zn(CH_3_COO)_2_, ZnSO_4_·7H_2_O and ZnCl_2_ through precipitation method. Hexamine was added to the solutions at 80°C for 6 h. Hexagonal pillar-like shape was obtained when NO_3_^−^ anion was used, the dumbbell-shaped particle was seen for CH_3_COO^−^ and hexagonally faceted plate-shaped particles were obtained for both SO_4_^−^ and Cl^−^ anions. Kaenphakdee et al. (2022) prepared ZnO using Zn(CH_3_COO)_2_ and Zn(NO_3_)_2_·6H_2_O. Precipitation method was used in which monoethanolamine in 2-methoxy ethanol was added for CH_3_COO^−^ anion precursor and hexamethylenetetramine in H_2_O for NO_3_^−^ anion precursor at 90°C for 2 h. These resulted in the aggregation of particles which yielded about 400–500 nm for CH_3_COO^−^ anion precursor and submicron rod-shaped particle at about 3 µm in length and 500 nm in diameter for NO_3_^−^ anion precursor. Kathalingam et al. (2015) synthesized various morphologies of ZnO by varying the zinc precursors (Zn(NO_3_)_2_·6H_2_O and Zn(CH_3_COO)_2_) the precursor’s concentration (10 mM- 0.5 M) as well as the preparation method. It was found that ZnO using NO_3_^−^ anion precursor shows spherical (45 nm), rod-like (35 nm), plate-like (120 nm), needle-like (32 nm), tube-like (35 nm) ZnO particles. The concentration of the precursor solution was varied leading to different morphologies as well. ZnO particles using CH_3_COO^−^ anion shows rod-like (15 nm) and wire-like structures (20 nm). Ozel et al. (2016) prepared ZnO particles using Zn(NO_3_)_2_·6H_2_O and ZnCl_2_ *via* hydrothermal method. NH_4_OH was added to the precursor solution at 100°C. Flower-like structure of ZnO was obtained with an average size of 5–7 µm when NO_3_^−^ anion precursor was used while rod-like ZnO was attained when Cl^−^ anion was used. Dey et al. (2021) reported on the precursor-dependent nanostructures of ZnO. Zn(CH_3_COO)_2_, Zn(NO_3_)_2_·6H_2_O and, ZnCl_2_ were used in the hydrothermal synthesis of ZnO at 120°C. Various morphologies of ZnO were obtained: nano-pencil, nanorods, and no defined shape for CH_3_COO^−^, NO_3_^−^, and Cl^−^ anions, respectively.

Different morphologies of CeO_2_ were observed as reported by Wu et al. (2008) CeCl_3_·7H_2_O and Ce(NO_3_)_3_·6H_2_O precursors were used in the hydrothermal reaction. The reaction was carried out at 140°C for 48 h producing CeO_2_ nanorods (15–25 nm in diameter and length up to a few micrometers) and CeO_2_ nanocubes (8–30 nm) for Cl^−^ and NO_3_^−^ anions, respectively. Kumar et al. (2017) prepared mesoporous CeO_2_ using CeCl_3_·7H_2_O, Ce(NO_3_)_3_·6H_2_O, (NH_4_)_2_Ce(NO_3_)_6_ and Ce(CH_3_COO)_3_ through hydrothermal reaction. The reaction was carried out at different conditions for each precursor used. L-glycine and Na_2_(CO_2_)_2_ solution were prepared and added to CeCl_3_ solution and hydrothermally heated at 160°C. In the case of Ce(CH_3_COO)_3_, Hexadecylamine in ethanol was added to the solution and stirred at room temperature. It was then placed in an oven at 60°C for 2 days. For Ce(NO_3_)_3_, a mixture of CTAB and NaOH was added to Ce(NO_3_)_3_ solution and stirred at 90°C and aged at 60°C for 12 h. Acryl amide, glucose, ammonia solution were added to (NH_4_)_2_Ce(NO_3_)_6_ solution and it was stirred at room temperature for 5 h. Transamidation of acetamide with N-octylamine was carried out and investigated using the CeO_2_ produced from these methods. It was found that CeO_2_ with a rod-like structure produced the highest conversion of acetamide.

Samiee and Goharshadi (2012) reported on the effects of different precursors on the properties of CeO_2_ in which CeO_2_ was prepared using Ce(NO_3_)_3_·6H_2_O and (NH_4_)_2_Ce(NO_3_)_6_ in a microwave-assisted synthesis. It was found that CeO_2_ synthesized using Ce(NO_3_)_3_·6H_2_O showed cubic-shaped particles with an average particle size of 7 nm. Similarly, CeO_2_ synthesized using (NH_4_)_2_Ce(NO_3_)_6_ was also showed cubic structure with an average particle size of about 3 nm. Aneggi et al. (2014) reported on the shape-dependent activity of CeO_2_ in soot combustion. Hydrothermal method was used to synthesize CeO_2_ in a basic condition using NaOH. Two different precursors were used namely, Ce(NO_3_)_3_·6H_2_O and CeCl_3_·7H_2_O in the synthesis producing CeO_2_ nanocubes and nanorods, respectively. The high stability of CeO_2_ for the catalytic combustion of chlorobenzene was synthesized using various cerium precursors (Zhang et al., 2021). Ce(NO_3_)_3_·6H_2_O, Ce(CH_3_COO)_3_, CeCl_3_·7H_2_O, and Ce(SO_34_)_3_·8H_2_O were used in hydrothermal synthesis at 180°C. It was observed that CeO_2_ synthesized from Ce(NO_3_)_3_·6H_2_O, Ce(CH_3_COO)_3_, CeCl_3_·7H_2_O and Ce(SO_34_)_3_·8H_2_O show rod-like (5–11 nm in diameter and 40–250 nm in length), lamellar structured particles (3–11 nm), a series of small spherical particles (5–23 nm) and strip structured particles (70–75 nm in width and 70–950 nm in length), respectively. It was found that rod-like CeO_2_ showed an increase in soot combustion activity.

Zhu et al. (2020) synthesized CeO_2_ using Ce(NO_3_)_3_·6H_2_O and CeCl_3_·7H_2_O in hydrothermal reaction for photocatalytic CO_2_ reduction. The synthesis was carried out at 140 and 180°C producing CeO_2_ nanocubes of about 30 nm length and nanorod of 200–400 nm in length and 20 nm in diameter when Ce(NO_3_)_3_·6H_2_O and CeCl_3_·7H_2_O were used, respectively. It was observed that CeO_2_ nanorods showed efficient photocatalytic CO_2_ reduction. Feng *et al.* reported on highly reducible nanostructured CeO_2_ for CO oxidation (Feng et al., 2018). Hydrothermal synthesis reaction was carried out using Ce(NO_3_)_3_·6H_2_O and CeCl_3_·7H_2_O at 110 and 160°C, respectively. Tube-like CeO_2_ was obtained with an average diameter of 30–70 nm and 1–5 µm in length for Ce(NO_3_)_3_·6H_2_O. Meanwhile, rod-like CeO_2_ at about 300 nm to 1 µm in length and 20–40 nm in diameter was observed for CeCl_3_·7H_2_O. The authors found that rod-like CeO_2_ exhibited the highest activity. Aboul-Gheit et al. (2014) prepared shape-dependent nano-TiO_2_ for the photodegradation of black b dye in water. TiO_2_ was synthesized using TiCl_4_ and Ti(OCH(CH_3_)_2_)_4_ *via* precipitation method. Semisphere particles of about 20 nm were obtained when TiCl_4_ was used whereas for the case of Ti(OCH(CH_3_)_2_)_4_, highly agglomerated CeO_2_ particles were obtained. Singh et al. (2017) synthesized TiO_2_ *via* sol-gel method using K_2_TiO(C_2_O_4_)_2_·2H_2_O and Ti(OCH(CH_3_)_2_)_4_. NH_4_OH was added to K_2_TiO(C_2_O_4_)_2_·2H_2_O solution and stirred at room temperature and diethanolamine was added in the Ti(OCH(CH_3_)_2_)_4_ solution. Irregular spherical to a mixture of platelet-shaped CeO_2_ (11–53 nm) and spherical (29–58 nm) for K_2_TiO(C_2_O_4_)_2_·2H_2_O and Ti(OCH(CH_3_)_2_)_4_, respectively.

Influence of different anions precursors on the morphologies of Co_3_O_4_ was reported by Hussain et al. (2014) Co(NO_3_)_2_·6H_2_O, CoCl_2_·6H_2_O, Co(CH_3_COO)_2_·4H_2_O and CoSO_4_·7H_2_O were used in the synthesis in a low temperature aqueous chemical growth. It was found that the synthesized Co_3_O_4_ showed a honeycomb-like, network of nanowires, grass-like and nanosheets when Co(NO_3_)_2_·6H_2_O, CoCl_2_·6H_2_O, Co(CH_3_COO)_2_·4H_2_O and CoSO_4_·7H_2_O were used, respectively. Various Fe precursors of Fe were used to produce Fe_2_O_3_ as reported by Guru et al. (2016) Microwave synthesis was used at 100°C by mixing ethylene glycol. NaOH, CTAB, and Fe precursors namely: Fe(NO_3_)_3_·9H_2_O, FeSO_4_·7H_2_O, Fe_2_(SO_4_)_3_·H_2_O and FeCl_3_·6H_2_O. For all cases, spherical particles were obtained in which the average particle sizes were in the range of 19–80 nm. In another report, α-Fe_2_O_3_ was synthesized hydrothermally from three different Fe sources: Fe(NO_3_)_3_·9H_2_O, FeCl_3_·6H_2_O and Fe(SO_4_)_2_·6H_2_O (Benhammada et al., 2020). Similarly, for all cases, spherical particles were observed giving an average particle size in the range of 80–110 nm (Sanjini et al., 2017). Microwave synthesized CuO NPs showed various morphologies when three different precursors were used. Spherical-shaped CuO NPs were obtained for the case of CuCl_2_, needle-shaped CuO NPs were obtained for the case of Cu(NO_3_)_2_, and spherical particles for the case of CuSO_4_.

**FIGURE 2:**
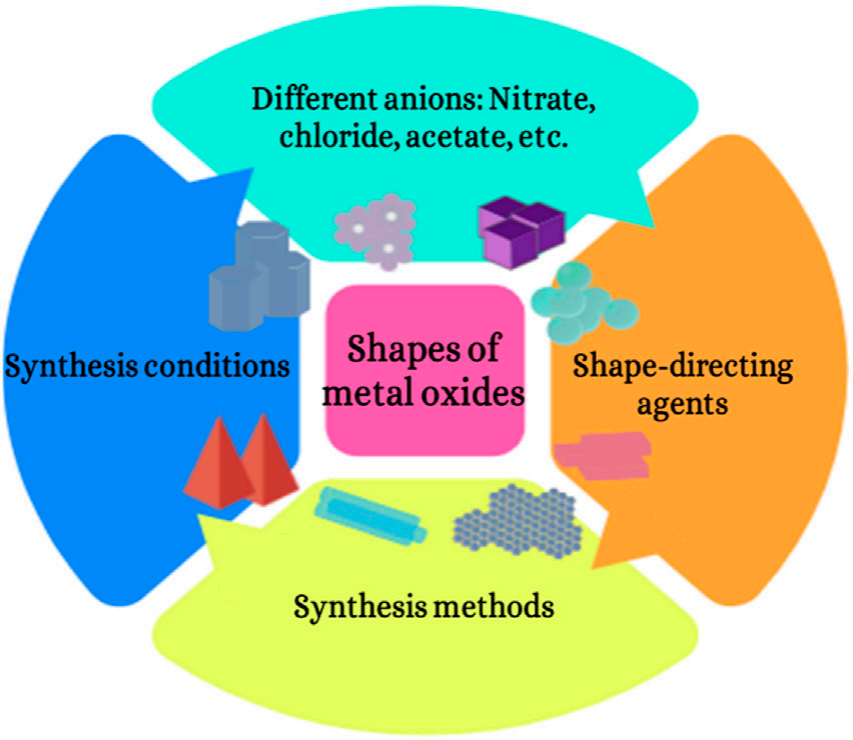
Effect of synthesis conditions on the morphologies of metal oxides.

Counter anions have different abilities to electrostatically stabilize individual nanoparticles into isolated highly crystalline solids during the full course of the reaction as stated by Pourrahimi et al. (2014) In general, the formation of spherical particles is thermodynamically more favorable (Khodashenas and Ghorbani, 2019). Hence, spherical particles have mainly been observed and obtained in the literature. It is well known that the nucleation and growth of nanostructures can be achieved using stabilizing agents with desired thermodynamic and kinetic control. The shape-selectivity of a semiconductor is usually achieved by additional shape-directing agents. These agents absorb preferentially on specific crystallographic planes leading to the change of direction and rate of crystal growth (Jain et al., 2019). Moreover, synthesis methods also play a role in the shape-selectivity of a semiconductor (Figure 2). Although there are many reports on the role of various additives in controlling crystal growth, there are only a few studies reported the influence of inorganic counter ions in shape-selective growth of metal oxide without the involvement of agents (Siegfried and Choi, 2005).

In the case of a typical synthesis of metal oxides using Cl^−^ counter anion precursor, the final shape of a metal oxide (MO) is generally rod-like. This has been illustrated in many literatures as can be seen in Table 1. In general, when Cl^−^ counter anion precursor is used in the synthesis with NaOH, it forms M(OH)_3_ (M = Zn, Ce, Ti, Co, Fe, and Cu) in which rod-like structure has been obtained. During the dehydration and oxidation process, the rod-like shape is not changed except for the size. It can be said that nanorod-like geometry is the intrinsic formation of the case of Cl^−^ (C. Nehru et al., 2012; Kaenphakdee et al., 2022; Dey et al., 2021). Apart from that, Cl^−^ directs the growth of particles into tube-like or wire-like particles. This is similar to rod-like structure however, the synthesis conditions such as temperature, acidity, and basicity as well as the use of capping agents leads to the distortion of the rod shape of particles (Samiee and Goharshadi, 2012; Zhu et al., 2020; Zhang et al., 2021).

**TABLE 1:** Various metal oxides synthesized using different precursors.

No	Materials	Precursors	Morphology and size	Phase	Applications	References
1	ZnO	i. Zn(CH_3_COO)_2_	i. Flower like (150–500 nm)	Hexagonal wurtzite	None	Panda et al. (2013)
		ii. Zn(NO_3_)_2_·6H_2_O	ii. Nanorods (150—500 nm)			
2	ZnO	i. Zn(NO_3_)_2_·6H_2_O	i. Mixture of nanoprisms and nanorods (length 18.91 nm and diameter 11.50 nm)	Hexagonal wurtzite	None	Gusatti et al. (2011)
		ii. ZnCl_2_	ii. Nanorods (23 nm)			
3	ZnO	i. Zn(NO_3_)_2_·6H_2_O	i. Star-shaped (500 nm)	Hexagonal wurtzite	None	Pourrahimi et al. (2014)
		ii. Zn(CH_3_COO)_2_·2H_2_O	ii. Cone-shaped (25 nm)			
		iii. ZnSO_4_·7H_2_O	iii. Petal-like (80–100 nm)			
		iv. ZnCl_2_	iv. Petal-like (80–100 nm)			
4	ZnO	i. Zn(CH_3_COO)_2_·2H_2_O	i. Dumbbell-like	Hexagonal wurtzite	None	van Rijt et al. (2020)
		ii. ZnCl_2_	ii. Hexagonally faceted plate-shaped			
		iii. Zn(NO_3_)_2_·6H_2_O	iii. Hexagonal pillar-shaped			
		iv. ZnSO_4_·7H_2_O	iv. Hexagonally faceted plate-shaped			
5	ZnO	i. Zn(CH_3_COO)_2_·2H_2_O	i. Aggregration particles (400—500 nm)	Hexagonal wurtzite	None	Pourrahimi et al. (2014)
		ii. Zn(NO_3_)_2_·6H_2_O	ii. Submicron rod-shaped (3 µm in length and 500 nm in diameter)			
6	ZnO	i. Zn(CH_3_COO)_2_·2H_2_O	i. Rod-like and wire-like (15—20 nm)	Hexagonal wurtzite	None	Kathalingam et al. (2015)
		ii. Zn(NO_3_)_2_·6H_2_O	ii. Spherical, rod-like, plate-like, needle-like and tube like (22–120 nm)			
7	ZnO	i. Zn(NO_3_)_2_·6H_2_O	i. Rod-like (0.5–1 µm)	Hexagonal wurtzite	None	Ozel et al. (2016)
		ii. ZnCl_2_	ii. Flower like (5–7 µm)			
8	ZnO	i. Zn(CH_3_COO)_2_·2H_2_O	i. Nanopencil	Hexagonal wurtzite	None	Dey et al. (2021)
		ii. Zn(NO_3_)_2_·6H_2_O	ii. Nanorods			
		iii. ZnCl_2_	iii. No defined shape			
9	CeO_2_	i. CeCl_3_·7H_2_O	i. Nanorods (15–25 nm diameters and lengths up to a few micrometers)	-	None	Wu et al. (2008)
		ii. Ce(NO_3_)_3_·6H_2_O	ii. Cube-like (8–30 nm)			
10	CeO_2_	i. CeCl_3_·7H_2_O	Mesoporous	Cubic	Transmidation of acetamide	Kumar et al. (2017)
		ii. Ce(NO_3_)_3_·6H_2_O				
		iii. Ce(CH_3_COO)_3_·6H_2_O				
		iv. (NH_4_)_2_Ce(NO_3_)_6_				
11	CeO_2_	i. Ce(NO_3_)_3_·6H_2_O	i. Cubic (7.08 nm)	Cubic	None	Samiee and Goharshadi, (2012)
		ii. (NH_4_)_2_Ce(NO_3_)_6_	ii. Cubic (3.37 nm)			
12	CeO_2_	i. Ce(NO_3_)_3_·6H_2_O	i. Nanocubes	Cubic	Soot combustion	Aneggi et al. (2014)
		ii. CeCl_3_·7H_2_O	ii. Nanorods			
13	CeO_2_	i. Ce(CH_3_COO)_3_	i. Lamellar (3–11 nm)	Cubic	Combustion of chlorobenzene	Zhang et al. (2021)
		ii. Ce(SO_4_)_3_·8H_2_O	ii. Almost spherical (5–23 nm)			
		iii. Ce(NO_3_)_3_·6H_2_O	iii. Nanorods (5–11 nm diameter and length 40–250 nm)			
		iv. CeCl_3_·7H_2_O	iv. Strip-like structure (70–75 nm			
14	CeO_2_	i. CeCl_3_·7H_2_O	i. Nanorod (200–400 nm length and 20 nm diameter)	-	CO_2_ photoreduction	Zhu et al. (2020)
		ii. Ce(NO_3_)_3_·6H_2_O	ii. Nanocubes (30 nm)			
15	CeO_2_	i. CeCl_3_·7H_2_O	i. Tube-like (1–5 µm length- 30–70 nm diameters)	Cubic	CO oxidation	Feng et al. (2018)
		ii. Ce(NO_3_)_3_·6H_2_O	ii. Rod-like (length of 300 nm to 1 µm and diameters of 20–40 nm)			
16	TiO_2_	i. TiCl_4_	Semisphere (20 nm)	Anatase	Photodegradation of black b dye	Aboul-Gheit et al. (2014)
		ii. Ti(OCH(CH_3_)_2_)_4_				
17	TiO_2_	i. K_2_TiO(C_2_O_4_)_2_·2H_2_O	i. Irregular spherical and platelet-like (11–53 nm)	Anatase	None	Singh et al. (2017)
		ii. Ti [OCH(CH_3_)_2_]_4_	ii. Spherical with agglomeration (29–58 nm)			
18	Co_3_O_4_	i. CoCl_2_·2H_2_O	i. Network of nanowires	Cubic	pH sensor	Hussain et al. (2014)
		ii. Co(NO_3_)_2_·2H_2_O	ii. Honey-comb like			
		iii. (CH_3_COO)_2_CO·4H_2_O	iii. Grass-like			
		iv. CoSO_4_·7H_2_O	iv. Nanosheets			
19	Fe_2_O_3_	i. FeSO_4_·7H_2_O	i. Spherical (19.4–46.7 nm)	-	None	Guru et al. (2016)
		ii. Fe_2_(SO_4_)_3_·H_2_O	ii. Spherical (29.1–67.6 nm)			
		iii. Fe(NO_3_)_3_·9H_2_O	iii. Spherical (29.1–40.8 nm)			
		iv. FeCl_3_·6H_2_O	iv. Spherical (29.1–80 nm)			
20	Fe_2_O_3_	i. FeCl_3_·6H_2_O	i. Spherical (110 nm)	Rhomboedral hematite	Thermal decomposition of cellulose	Benhammada et al. (2020)
		ii. Fe(NO_3_)_3_·9H_2_O	ii. Spherical (90 nm)			
		iii. Fe(SO_4_)_2_·6H_2_O	iii. Spherical (80 nm)			
21	CuO	i. CuCl_2_	i. Spherical	Monoclinic	Methylene blue degradation	Sanjini et al. (2017)
		ii. CuNO_3_	ii. Needle shape			
		iii. CuSO_4_	iii. Spherical			

Interestingly, when NO_3_^−^ salts were introduced, the morphology of the metal oxides was directed into faceted shaped MO (cube, plate-like, hexagonal, honeycomb, etc.) (C. Nehru et al., 2012; Jayakumar et al., 2022; Wang et al., 2010; Panda et al., 2013; Zhu et al., 2020). Typically, metal oxides form polyhedral-kind of shape and in order to tune the surface free energies and induce the anisotropic growth of well-shaped nanostructures, adscititious surfactants are required in which this is the case for most of the reported shapes (Table 1) (Yang and Gao, 2006). However, a dissolution-recrystallization process under the strong basic condition would influence the production of cube-like or faceted metal oxides particles (Yang and Gao, 2006).

Pourrahimi et al. (2014) has conducted studies on the probable “shielding effect” of different counter anions on the particle stabilization. It was found that both Cl^−^ and NO_3_^−^ ions showed inability to stabilize the particles. Furthermore, nitrate-based precursor has shown to produce smaller particles which was aimed to grow specific directional morphologies in hydroxide solutions (Cho et al., 2008). On the other hand, CH_3_COO^−^ ions has the strong ability to stabilize as it has been suggested to originate from strong uni- and bi-dentate oxygen coordination bonding of the acetate ions to individual metal atoms, or parallel bridging of the two oxygen atoms in the CH_3_COO^−^ ions to positively charged metal atoms of the particles (Sun et al., 2007; Segets et al., 2011; Pourrahimi et al., 2014). Moreover, Nicholas et al. (2012) stated that, partially positively charged methyl functional unit of the CH_3_COO^−^ ions associated with the insufficiently condensed negatively charged metal hydroxide which therefore suggesting the stabilization of growing nanoparticles probably derived from a formed amphiphilic capping layer around the particle (Pourrahimi et al., 2014).

Spherical-shaped CeO_2_ has shown high efficiency in photocatalysis activities due to its small particle size and high surface areas (Sanjini et al., 2017; Benhammada et al., 2020). However, nanoshaped CeO_2_ (cube, rod, hexagonal, etc.) are evident to have effects on photocatalysis activities. This is because nanoshaped CeO_2_ enabled the study of the correlation between exposed surfaces and photocatalytic activities. Anneggi *et al.* proposed that {100}/{110} exposed surfaces are more reactive in photocatalysis activities, particularly on CO oxidation. This observation can be seen in many studies (Kumar et al., 2017; Feng et al., 2018; Zhu et al., 2020; Zhang et al., 2021).

## Anion Directed Synthesis of Chalcogenides

**FIGURE 3:**
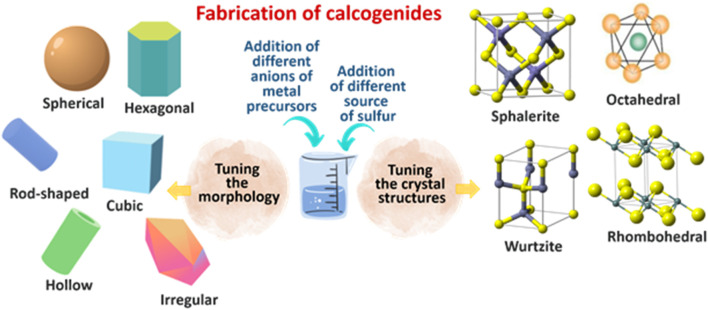
Different morphologies and crystal structures of chalcogenides synthesized using different anion metal precursors and different sources of sulfur.

Chalcogenides are narrow-band gap semiconductors consisting of at least one chalcogen anion (sulphide, selenide, or telluride) and at least one more electropositive element (Khan and Khan, 2021; Rahman and Khan, 2021). Unlike metal oxide, researchers have widely explored varying the sulfur precursors for chalcogenides instead of varying the anions of the metal precursors. Table 2 shows some of the reported works on varying the precursors of selected chalcogenides. Over the last decades, many preparation routes have been developed for the synthesis of chalcogenides with different morphologies, particle sizes, and crystal structures that can be obtained from different raw materials through different synthetic pathways (Figure 3). Various authors have investigated the effect of anion on the morphology, particle size, and crystal structure of different chalcogenides. For instance, Gaur and Jeevanandam (2015) investigated the effect of anions (acetate, chloride, nitrate, and sulfate) in diphenyl ether and in solid-state that leads to the formation of CdS nanoparticles with different morphologies. The CdS nanoparticles derived from solid-state thermal decomposition of the cadmium-thiourea complexes with acetate, chloride, nitrate, and sulfate ions exhibited spheres, nanotubes, nanoflowers, and irregular morphologies, respectively. On the other hand, thermal decomposition of the cadmium thiourea complexes with acetate, chloride, and nitrate ions in diphenyl ether results in CdS nanoparticles with microspheres, nanopyramids, and a mixture of nanoparticles and nanorods morphologies, respectively. Amongst the synthesized materials, CdS synthesized from cadmium acetate and thiourea *via* solid-state exhibited the highest photocatalytic crystal violet degradation of 99.2%.

**TABLE 2:** Summary of previous work on the effect of anions on the morphology, particle size, and crystal structure of various chalcogenides.

No	Materials	Metal precursors	Sulfur precursors	Morphology and size	Crystal phase	Application	References
1	CdS synthesized *via* thermal decomposition	Cadmium acetate	Thiourea	Cadmium acetate: spheres with diameter ∼100–200 nm	Hexagonal and cubic	Photocatalytic degradation of crystal violet	Gaur and Jeevanandam, (2015)
		Cadmium chloride		Cadmium Chloride: nanotubes with diameter ∼70–100 nm			
		Cadmium nitrate		Cadmium nitrate: nanoflowers with diameter ∼150–200 nm			
		Cadmium sulfate		Cadmium sulfate: irregular morphologies			
2	MoS_2_ synthesized *via* silica sol method	(NH_4_)_6_Mo_7_O_24_·4H_2_O	Thiourea	Thiourea: nanowires with high crystallinity	2H-MoS_2_	Hydro-deoxygenation	Zhang et al. (2017)
			L-cysteine	L-cysteine: nanowires with poor crystallinity			
3	ZnS synthesized *via* spray pyrolysis	ZnCl_2_	Thiourea	Small clusters with average size of 80–100 nm	Wurtzite	-	Zeng et al. (2013)
			Thioacetamide				
4	CdS/MoS_2_ synthesized *via* hydrothermal method	CdCl_2_·2H_2_O	Thiourea	Thiourea: granular in shape	Both the cubic and hexagonal phases of CdS were present	Photocatalytic degradation of methylene blue	Wang et al. (2018)
		Na_2_MoO_4_·2H_2_O	L-cysteine	L-cysteine: spherical porous structure			
			Thioacetamide	Thioacetamide: rod-like and flower-like Thiourea: cauliflower-like morphology with an average diameter of 0.8–1 μm			
5	ZnS synthesized *via* hydrothermal method	Zn(CH_3_COO)_2_·6H_2_O	Thiourea, Sodium sulfide nonahydrate, Thioacetamide	Sodium sulfide: rice grain-shaped microstructures with size of 15–20 mm long, 1–2 mm thick and 2–5 mm wide	Cubic	Laser-induced reduction of Cr(VI)	Kim et al. (2016)
				Thioacetamide: roughly hedge apple-like shape with an average diameter of approximately 1–2 μm.			
6	ZnS synthesized via chemical bath deposition	ZnSO_4_	Thiourea	-	Wurtzite	-	Kozhevnikova et al. (2020)
		ZnCl_2_	Thioacetamide		Sphalerite		
			Sodium thiosulfate	FeSO_4_·7H_2_O: short nanorods having length up to 500 nm and diameter within 40–100 nm			
			Sodium sulfide				
7	FeS_2_ synthesized *via* solvothermal method	FeSO_4_·7H_2_O FeCl_3_	Thiourea	FeCl_3_: large nanowires (>90%) along with some micro-rods	Cubic pyrite	-	Kar and Chaudhuri, (2004)
		Fe(NO_3_)_3_9H_2_O		Fe(NO_3_)_3_·9H_2_O: nanowires with diameter in the range 40–60 nm and length up to tens of μm			
				Thioacetamide: Ni_3_S_2_ nanorods and small MoS_2_ nanosheets			
8	MoS_2_/Ni_3_S_2_ synthesized *via* hydrothermal method	Na_2_MoO_4_2H_2_O	Thioacetamide L-cysteine	L-cysteine: irregular nanoparticles	-	Electro-chemical measurements	Liu et al. (2018)
			Thiourea	Thiourea: nanowires with diameters of about 200–300 nm			
9	CdS synthesized *via* hydrothermal method	Cd(NO_3_)_2_4H_2_O	Thiourea Thioacetamide L-cysteine	Thiourea: dendritic-like	Thiourea and L-cysteine: hexagonal	Photocatalytic hydrogen production	Li et al. (2018)
				Architecture with diameter and length of the trunk are 0.3 and 2.5 μm, respectively	Thioacetamide: mixture of hexagonal and metastable cubic CdS		
				Rod-like Morphology			
10	CdS synthesized *via* solvothermal method	Cd(NO_3_)_2_4H_2_O	Thiourea	Nanorods with diameter of around 10–20 nm	Zinc blende	Photocatalytic degradation of methylene blue, methyl orange, safranin O, rhodamine B and remazol brilliant yellow	Malik et al. (2016)
				Flower-like morphology with the diameter of around 30–40 nm	Wurtzite		
		Cd(CH_3_COO)_2_·2H_2_O		Elemental sulphur: irregular structures at the base of the nanobars			
11	Ag-modified CdS synthesized *via* solvothermal method	CdCl_2_	Elemental sulphur, thiourea and L-cysteine	Thiourea: spherical-like structures forming globular aggregates	Hexagonal	Photocatalytic production of H_2_	Soto Morillo et al. (2020)
		Ag(CH_3_COO)		L-cysteine: filamentous structures and lamellar aggregates			
12	ZnS synthesized *via* hydrothermal method	Zinc acetate	Thiourea	Zn(NO_3_)_2_ and thiourea: ∼400 nm nanobelts	Zn(NO_3_)_2_ and thiourea: wurtzite	Photocatalytic degradation of methylene blue	Kanti Kole et al. (2014)
		Zinc nitrate	Sodium sulphide	Zn(CH_3_COO)_2_ and Na_2_S: spheroidal and cuboidal shaped ZnS with average size of average size ∼100–200 nm	Zn(CH_3_COO)_2_ and Na_2_S: Zinc blende		
13	CdS synthesized in a hot-paraffin matrix	Cadmium stearate	Tributyl-phosphine sulfide	Quantum dots with mean diameter of 3.67 (±0.27) nm	N Amorphous sphalerite structure	-	Yordanov et al. (2006)
			Elemental sulfur				
			Ammonium sulphide				
14	CdS synthesized via chemical precipitation	Cadmium nitrate	Hydrogen sulphide	Spherical quantum dots with particle size less than 10 nm	Wurtzite and zinc blende	Photocatalytic degradation of Acid Blue-29	Qutub et al. (2016)
			Sodium sulphide				

15	CuInS_2_	bis (2-hyroxyacetophenato) copper (II)	Thioacetamide	When carbon disulfide was used instead of thioacetamide in the formation of CuInS_2_ in ethylene glycol, irregular plate-like and bulky particles were achieved	Tetragonal	-	Sabet et al. (2013)
			Thiourea				
			L-cysteine				
			Carbon disulfide				
			Thiosemi-carbazide				
			Thioglycolic acid				
			Ammonium sulfide				
			Sodium sulfite				

In a different study, two morphologies of MoS_2_ were obtained by using thiourea and L-cysteine as sulfur sources (Zhang et al., 2017). Zhang *et al.* reported that MoS_2_ prepared by using thiourea had a petal-shaped structure, and the crystal size was larger while MoS_2_ prepared from L-cysteine had a loose structure, and the crystal size was smaller. They also reported that MoS_2_ prepared from thiourea exhibited better catalytic performance than that from L-cysteine in the hydrodeoxygenation reaction. Hydrothermal assisted synthesis of CdS/MoS_2_ using three different sulfur sources; thioacetamide, L-cysteine, and thiourea has been reported by Wang et al. (2018) Their results showed that the different sulfur sources induced differences in crystallization, morphology, elemental composition, and absorption in the UV–visible light region. Among the three sulfur sources, CdS/MoS_2_ prepared using thioacetamide showed excellent adsorption performance and the highest photocatalytic ability to degrade methylene blue with approximately 97% within 120 min under visible light irradiation, much higher than that achieved by CdS/MoS_2_ prepared using L-cysteine and thiourea.

Kim et al. (2016) have successfully controlled the morphology of ZnS by utilizing anionic precursors *via* a hydrothermal method for reduction of Cr(VI). The authors reported rate of nucleation is the main factor affecting the morphology variations, and it mainly depends on the rate of release of S^2-^ by the anionic thiourea, thioacetamide, and sodium sulfide precursors. When thiourea and thioacetamide are used as the sulfur sources, the rate of S^2-^ release is slow compared to that achieved with sodium sulfide. The rate of release of S^2-^ from thiourea, in particular, is very slow in comparison to that from thioacetamide because of the strong coupling between the -NH_2_ group and the nanoparticles. The ZnS nanostructures prepared using thiourea as a sulfur source had a cauliflower-like morphology with an average diameter of 0.8–1 μm. When sodium sulfide was used as the sulfur source, rice grain-shaped microstructures were produced while thioacetamide produces hedge apple-like shape with an average diameter of 1–2 μm.

In another study, Kozhevnikova et al. (2020) have successfully synthesized ZnS using the chemical bath deposition method. In this study, they have used different sources of sulfur including thiourea, thioacetamide, sodium thiosulfate, and sodium sulphide. All the synthesized ZnS exhibited wurtzite and sphalerite ZnS structures. In addition to this, they have also reported that the chemical nature and initial concentrations of ZnSO_4_ and ZnCl_2_ salts have no significant effect on particle size, phase composition, and crystal structure of ZnS colloids. FeS_2_ with different morphologies have been successfully synthesized *via* solvothermal method as reported by Kar and Chaudhuri (2004) They found that the anions of the iron source (FeSO_4_·7H_2_O, FeCl_3_ and Fe(NO_3_)_3_·9H_2_O), temperature, and the molar concentrations of the precursors in the solvent play an important role in controlling the morphology of the FeS_2_. When FeSO_4_·7H_2_O was used as the iron source, short nanorods having lengths up to 500 nm and diameter within 40–100 nm were produced. When FeCl_3_ was used, large FeS_2_ nanowires along with some micro-rods were observed. When Fe(NO_3_)_3_·9H_2_O was used as the precursor, uniform nanowires with diameters in the range 40–60 nm and length up to tens of μm.

Liu et al. (2018) reported that the different sources of sulfur in synthesizing MoS_2_/Ni_3_S_2_ heterostructure have a significant influence on its structures and morphologies. They reported that MoS_2_/Ni_3_S_2_ prepared from thioacetamide showed Ni_3_S_2_ nanorods and small MoS_2_ nanosheets while L-cysteine showed the formation of irregular nanoparticles. In addition, nanowires with diameters of about 200–300 nm were observed when MoS_2_/Ni_3_S_2_ prepared from thiourea. The thioacetamide-assisted synthesis of MoS_2_/Ni_3_S_2_ showed superior H_2_ evolution reaction activities due to the higher content of MoS_2_ and it exhibited a larger electrochemically active surface area which provides more active sites for the H_2_ evolution reaction. Li et al. (2018) have also reported the effects of these sulfur sources (thiourea, thioacetamide, and L-cysteine) on the properties of the resulted CdS including the crystal structure, morphology, and photocatalytic performance for H_2_ evolution reaction. Based on their study, CdS prepared using thiourea with hexagonal branched dendritic structure has the smallest interfacial electron transfer resistance and the most negative conduction band bottom, and consequently shows the highest H_2_ evolution reaction. CdS prepared using thioacetamide on the other hand exhibited a mixed phase of hexagonal and cubic which facilitated the recombination of photogenerated charge carriers that leads to a considerably lower H_2_ evolution performance in comparison to CdS synthesized using thiourea. Moreover, low crystallized hexagonal CdS nanoparticles with no specific morphology were observed for CdS prepared using L-cysteine as the source of sulfur showed the largest interfacial electron transfer resistance and this resulted in the lowest H_2_ evolution reaction.

Kanti Kole et al. (2014) have been successfully synthesized ZnS nanostructures of different morphologies, such as block-like, belt-like, spheroidal, and cuboidal shaped nanoparticles by using the simple hydrothermal technique. It has been shown that controlling the amount of sulphur precursor or utilizing different types and ratios of zinc and sulphur precursors can easily alter both the phase and morphology of ZnS nanostructures. They also reported that pure phase wurtzite ZnS nanobelts exhibited superior performance for the degradation of methylene blue dye with a degradation efficiency of 98% within 40 min of UV light irradiation. Different crystal structures of CdS nanoparticles prepared *via* chemical precipitation method using different sulfur sources ((NH_4_)_2_S, H_2_S, Na_2_S) have been reported by Qutub *et al.* Their group has studied the effect of different sulfur sources on the size of nanoparticles, respective band gaps, and crystalline structure. Based on their findings, a smaller particles size for CdS prepared using Na_2_S, followed by H_2_S and (NH_4_)_2_S was observed, and the quantization in the band gap was directly in correlation with decreased particle size effects. Moreover, a mixed-phase of wurtzite and zinc-blende was obtained for CdS synthesized H_2_S, while the pure phase of zinc-blende and wurtzite was obtained with Na_2_S and (NH_4_)_2_S, respectively. They also reported that CdS synthesized using Na_2_S with the addition of sodium hydroxide and methanol exhibited the highest activity and almost completely decolorized the derivative Acid Blue-29 under irradiation of visible light within 90 min. Tang et al. (2015) reported a one-pot synthesis of CuInS_2_ using different anions to engineer their morphology and crystal structure. CuInS_2_ having chalcopyrite, zinc blende, and wurtzite phases have been successfully synthesized by carefully selecting anions in metal precursors and manipulating reaction parameters such as reactant molar ratios and reaction temperature. They reported that CuInS_2_ nanoplates with a wurtzite-zinc blende polytypism structure are formed in the presence of Cl^−^ ions. Furthermore, they also reported that the optical absorption measurements of CuInS_2_ exhibited a strong dependence on the crystal structure and size.

Generally, the preparation methods and the conditions of synthesis are crucial factors for fabricating chalcogenides, and they possess a major role in the chemical as well as structural applications of chalcogenides. In addition to that, the influence of utilizing different anionic metal precursors and/or different sources of sulfur on the structural and morphological properties of chalcogenides was not largely reported in comparison to other semiconductors. Chalcogenides with controllable crystal structures and morphologies have potential applications in various areas as diverse as catalysis, plasmonics, sensing/imaging, spectroscopy, and medicine.

## Challenges During the Synthesis and Crystal Growth of Semiconductors

Properties of metal oxides have been considered to be dependent on the morphologies. However, in order to produce targeted shapes of a metal oxide, some agents should be employed in the synthesis. Therefore, metal oxides with different morphologies without the use of agents have become a major challenge. Fabrication of chalcogenides, in particular, can be quite challenging because of their stability. In addition to that, the selection of a suitable precursor is a crucial stage because it will not only have an influence on the physical properties of the materials but also its chemical and optical properties. Moreover, it is also important to avoid the use of toxic precursors, environmentally friendly solvents, keeping the reaction temperature close to room temperature, and also minimizing the quantities of generated by-products are great advantages that make the synthesis of metal oxides and chalcogenides outstanding.

## Future Prospects

Controlled crystal growth of semiconductors is crucial for activity efficiency in various applications (biological, environmental, and energy). The controlled crystal growth can be achieved by changing the anion precursor salts and keeping other conditions the same. However, to date, the reports on this matter are still less in number in which some research gaps are yet to be answered. The following are the future prospects that should be considered and addressed:

• Most of the syntheses using different anion precursors to produce different shapes require different synthesis conditions. In order to effectively study the role of anions, one should keep other conditions the same and vary the anion precursors only.
• Most syntheses and studies still require stabilizing and capping agents to aid the formation of different morphologies of a semiconductor.
• In-depth study of crystallographic properties of a semiconductor should be carried out to study the overall effect of different anions on a semiconductor.
• Deeper understanding of the growth mechanisms of the semiconductor *via* computational simulation would help the researchers to fabricate materials with desired properties more efficiently.

## Conclusion

Various morphologies of semiconductors (metal oxides and chalcogenides) have been obtained using different anion precursor salts through typical synthesis methods. Spherical particles are normally observed due to their thermodynamically favorable properties. However, by changing the anion precursor’s salts, the morphology of a semiconductor is affected accordingly. This can be said that the anions have some effects on the final forms of a semiconductor. Nevertheless, in-depth studies are required to investigate the effect of anions on the crystal growth of a semiconductor to get maximum efficiency for the fabricated particles.
